# The feasibility and efficacy of computer-assisted screw inserting planning in the surgical treatment for severe spinal deformity: a prospective study

**DOI:** 10.1186/s12893-022-01711-y

**Published:** 2022-07-09

**Authors:** Yiqi Zhang, Yong Hai, Jincai Yang, Peng Yin, Chaofan Han, Jingwei Liu, Lijin Zhou

**Affiliations:** 1 Department of Orthopedics, Beijing Chaoyang Hospital, Capital Medical University, GongTiNanLu 8#, Chaoyang District, 100020 Beijing, China; 2 Department of Orthopedics, Beijing Hospital, Peking University, DongDanDaHuaLu 1#, Dongcheng District, 100005 Beijing, China

**Keywords:** Spinal deformity, Computer-assisted, Screw placement, Software

## Abstract

**Background:**

The objective of the study was to explore the feasibility and efficacy of computer-assisted screw inserting planning (CASIP) in the surgical treatment for severe spinal deformity.

**Methods:**

A total of 50 patients participated in this prospective cohort study. 25 patients were allocated into CASIP group and 25 patients were in Non-CASIP group. The demographic data, radiological spinal parameters were documented and analyzed. Each pedicle screw insertion was classified as satisfactory insertion or unsatisfactory insertion based on Gertzbein-Robbins classification. The primary outcome was the accuracy of pedicle screw placement. The secondary outcomes were the rate of puncturing screws, estimated blood loss, surgical time, correction rate and other radiological parameters.

**Results:**

A total of 45 eligible patients completed the study. 20 patients were in CASIP group and 25 patients were in Non- CASIP group. The accuracy of pedicle screw placement in CASIP Group and Non-CASIP Group were (92.0 ± 5.5) % and (82.6 ± 8.3) % (P < 0.05), and the rate of puncturing screws were (0 (0–0)) % and (0 (0-6.25)) % (P < 0.05). The median surgical time were 280.0 (IQR: 260.0–300.0) min and 310 (IQR: 267.5–390.0) min in two group and showed significant statistic difference (P < 0.05).

**Conclusions:**

CASIP has good feasibility and can gain a more accurate and reliable instruments fixation, with which spine surgeons can make a detailed and personalized screw planning preoperatively to achieve satisfying screw placement.

## Introduction

Severe spinal deformity is one of the most challenging problems that spine surgeon needs to face, and it may lead to both mental and physical issues including cardiopulmonary disfunction, neurological deficits and poor self-image and cosmetic view [1, 2]. As the pedicle screw fixation technique develops, spinal deformity correction surgeries have witnessed a revolutionary change [3, 4]. With solid fixation provided by pedicle screws, high grade osteotomy such as vertebra column resection would be possible, and satisfying correction in radiography was achieved [5]. Nevertheless, studies have demonstrated abnormal morphology formation in spinal deformity, which would increase the risk of neurological deficits because of pedicle screws inserting malposition [6–8]. To achieve a satisfying and safe correction, solid instrumented fixation by the accurate pedicle screw inserting must be required, or it would cause a range of complications including neurologic complications [9].

Computer-assisted virtual surgical planning has been reported widely using in spine surgery [10–12], with which spine surgeons could observe the spine morphology clearly with a 3D spine model and personalized surgical plan would be achieved. However, we noticed that very little research about the technique has focused on spinal deformity. Considering that vertebral rotation and complex anatomy were common in spinal deformity, we presumed that computer-assisted screw inserting planning (CASIP) would provide a boundless application in improving the accuracy of pedicle screw placement. The purpose of the present study was to explore the feasibility and efficacy of CASIP in the surgical treatment for severe spinal deformity.

## Methods

### Patients selection

This was a prospective cohort study that contained severe spinal deformity patients in our institute from November 2021 to April 2022. Patients were divided into CASIP group and Non-CASIP group according to whether CASIP technique was applied before the correction surgery. All the demographics and radiological data were documented. Inclusion criteria were listed: (1) severe spinal deformity patients with Cobb angle of main curve > 80° [13]. (2) patients underwent posterior correction surgery with pedicle screws. Exclusion criteria were listed: (1) spine tumor, infection and trauma. (2) MRI demonstrated Chiari malformation, syringomyelia and other spinal abnormalities. (3) radiologic data incomplete. The surgery was performed by the same surgeon. The study was approved by the institutional review board of our institution and it was registered at Chinese Clinical Trial Registry with the unique identifier as ChiCTR2100053808 (29/11/2021). All the patients have provided written informed consent.

### Sample size

This was a pilot prospective study. There were no parameters referred to estimate the sample size, and Hertzog et al. [14] has shown that 10–20 subjects per group are adequate to evaluate the feasibility of a pilot study. Thus, we aimed to recruit 25 patients per group.

### 3D model establishment

Patient’s CT scan data were input with Digital Imaging and Communications in Medicine (DICOM) format (Siemens CT machine, SOMATOM Sensation 16, Siemens AG, Forchheim Germany), the thickness of the fault was 2 mm so as to make a suitable virtual whole spine. All the tomographic pictures of the patient were imported into Mimics Medical 21.0 (Materialise NV, Leuven, Belgium). Virtual 3D model was created for measurements and 3D printing with threshold of 226-3071HU.

### Screw inserting planning

**Fig. 1:**
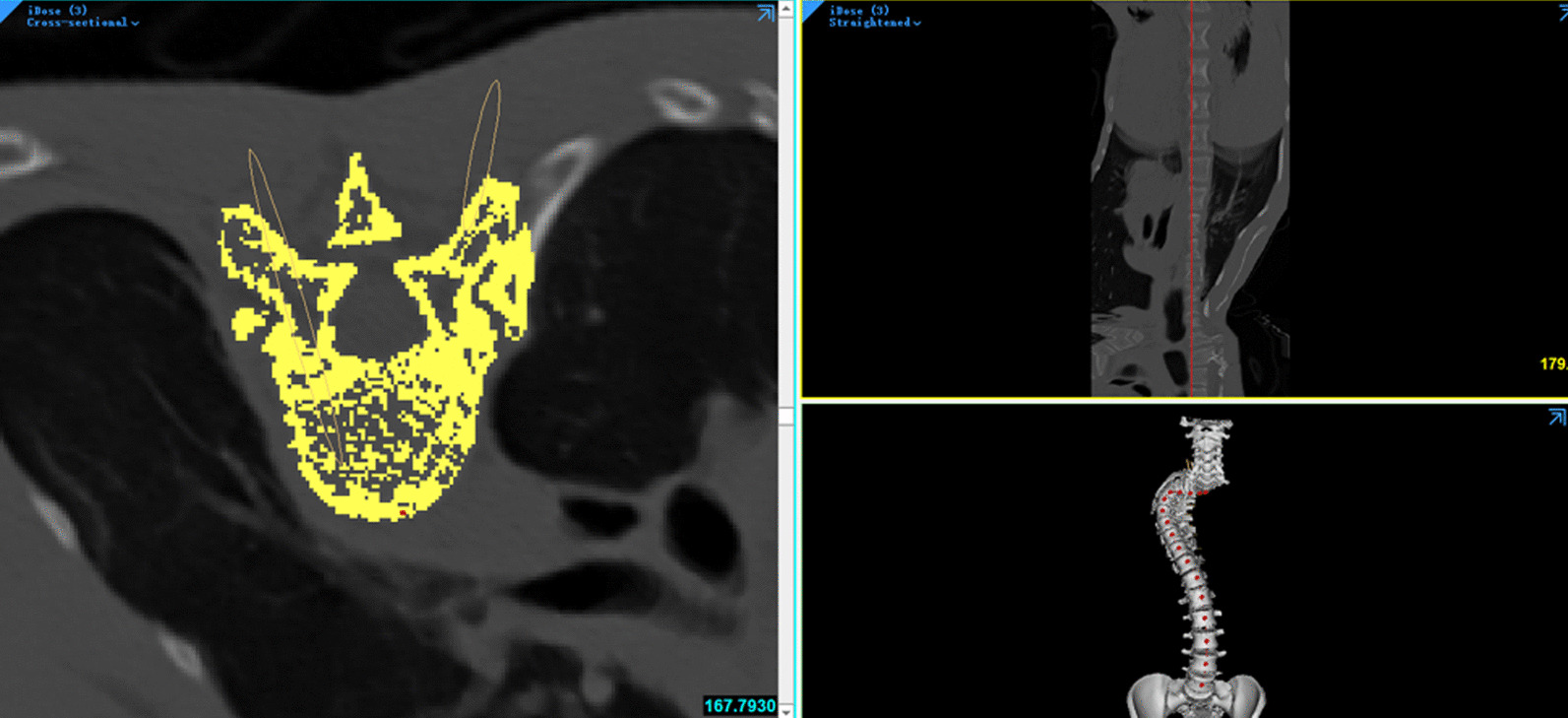
Illustration of reslicing CT data

**Fig. 2:**
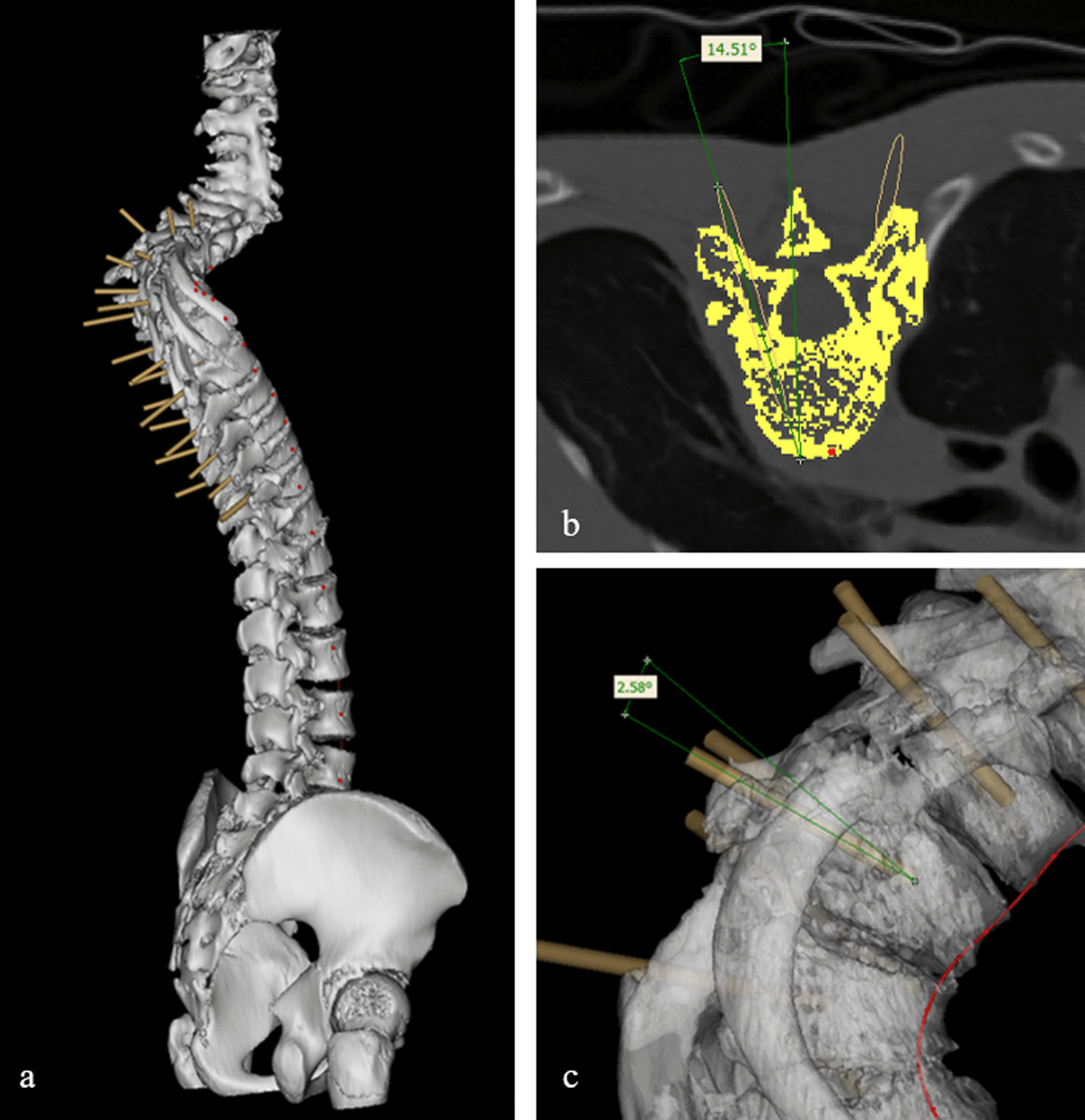
Preset screws were simulated by cylinders and measured in CASIP. **a** position check in 3D spine model; **b**, lateral angle measurement; **c** cephalic angle measurement

**Fig. 3:**
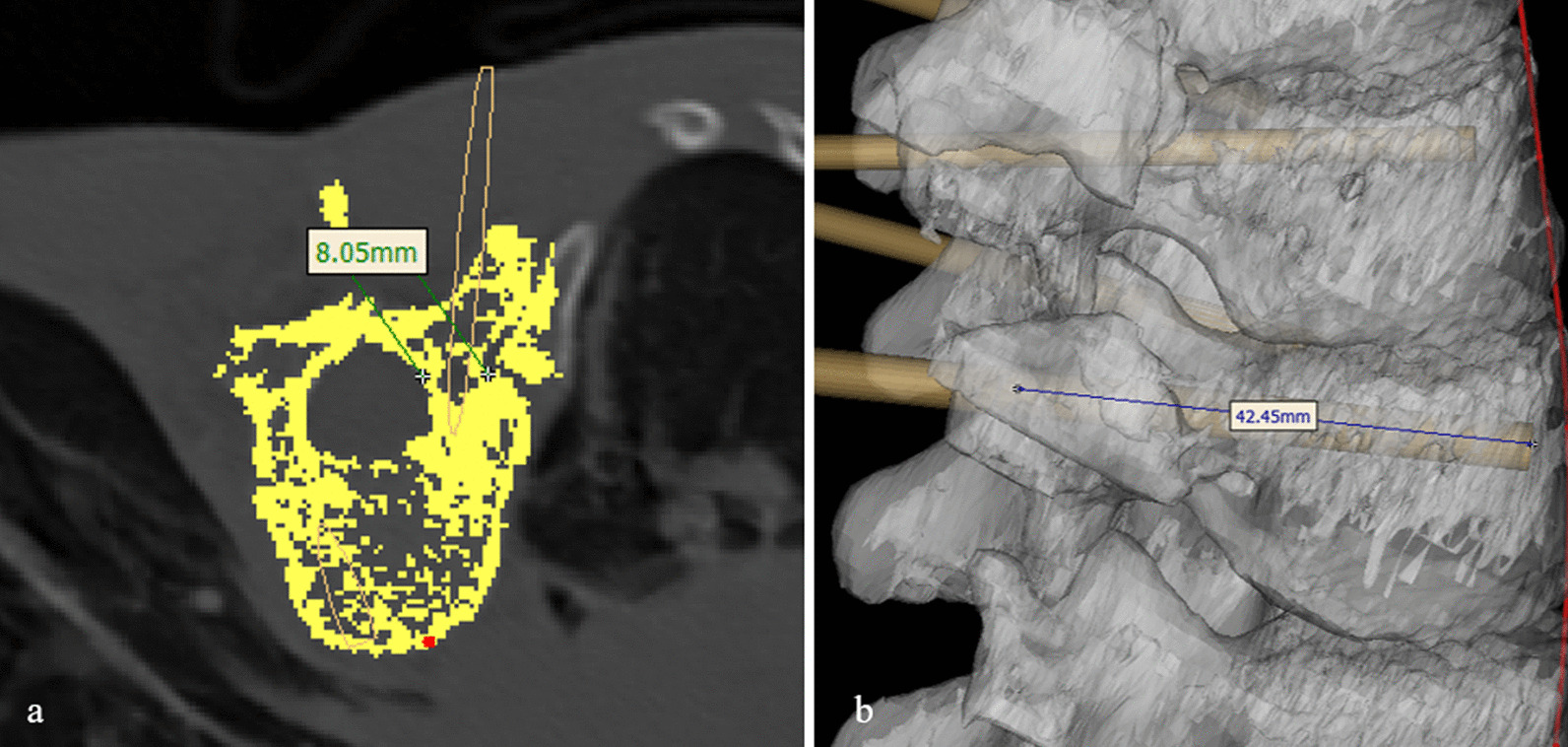
Size evaluation for preset screw. **a** width of the pedicle; **b** length of the screw

**Fig. 4:**
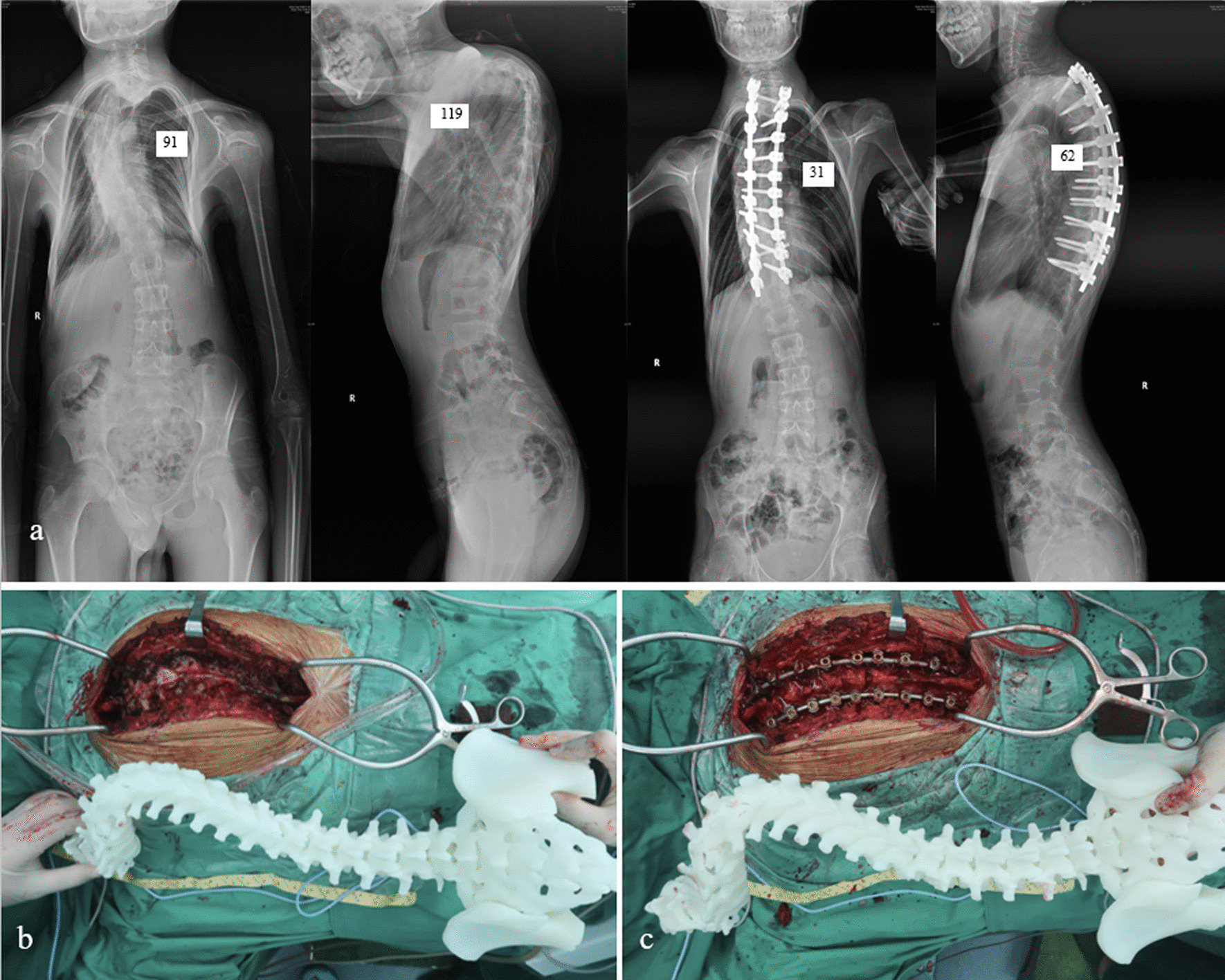
A 16-year-old male patient diagnosed as severe spinal deformity underwent correction surgery with CASIP from T2-T11. **a** preoperative and postoperative full spine X-ray. **b** intraoperative illustration of 3D printed spine model before screw placement. **c** intraoperative illustration of 3D printed spine model after screw placement

In 3D model, designed cylinder was inserted into vertebra to simulate pedicle screw via axial plane and sagittal plane with resliced data along the curve. Reslicing CT data was performed by selecting midpoint of the vertebra surface along the curve, then resliced images could demonstrate pedicle level horizontally (Fig. 1). Cylinders were checked and adjusted in 3D view (Fig. 2a) to guarantee accurate placement of simulated pedicle screws. Lateral angle and cephalic angle were measured and the data were applied during the surgery (Fig. 2b, c). The length and width of the preset screw were measured and recorded (Fig. 3). A 3D printed spine model was established as an intuitive reference to match the data during the surgery for each patient in CASIP group (Fig. 4). Those with narrow pedicles or abnormal vertebra morphology hard to recognized would be required a 3D-printed template for screw inserting.

For CASIP group, patients with planned implementation rate of planned screws < 80% would be excluded in the final analysis. In addition, in CASIP group, only implemented planned screws were analyzed. The study was performed based on per-protocol analysis [15].

### Outcome assessments

The primary outcome was the accuracy of pedicle screw placement. All patients underwent full spine CT scan before discharge and all pedicle screws were recorded and accuracy were evaluated according to Gertzbein–Robbins classification (Grade A : no violation of any cortex pedicle; Grade B–D: penetrates the cortical layer of the pedicle < 2 mm, 4 mm, 6 mm, respectively; Grade E: breach the cortex pedicle in any direction > 6 mm or outside the pedicle)) [16, 17]. Grade A and B were defined as satisfactory insertion, and C to E were unsatisfactory insertion. The accuracy of pedicle screw placement for each patient was defined as satisfactory screws/ total screws. The secondary outcomes were as follows: Cobb angle of main curve, focal kyphosis (FK), thoracic kyphosis (TK), lumbar lordosis (LL) and sagittal vertical axis (SVA), correction rate of main curve, correction rate of FK, surgical time and estimated blood loss. The rate of puncturing screws of each patient was calculated by screws that punctured the vertebral wall / total screws.

All the measurements were performed by two independent orthopedic doctors who were blind to the allocation, any discrepancy was resolved by their discussion and re-evaluation.

### Statistical analysis

Statistical analysis of all data was calculated by SPSS Statistics 20 (IBM Corp, Armonk, New York, United States). Continuous variables were described as mean ± SD or medians (IQRs) for normally distributed variables and abnormally distributed variables, and categorical variables were described as proportions. Independent Student t test or the Mann–Whitney U test was utilized to assess differences between two groups. Chi-square test was used to compare attributes data between groups, and P < 0.05 was defined as statistical significance.

## Results

**Fig. 5:**
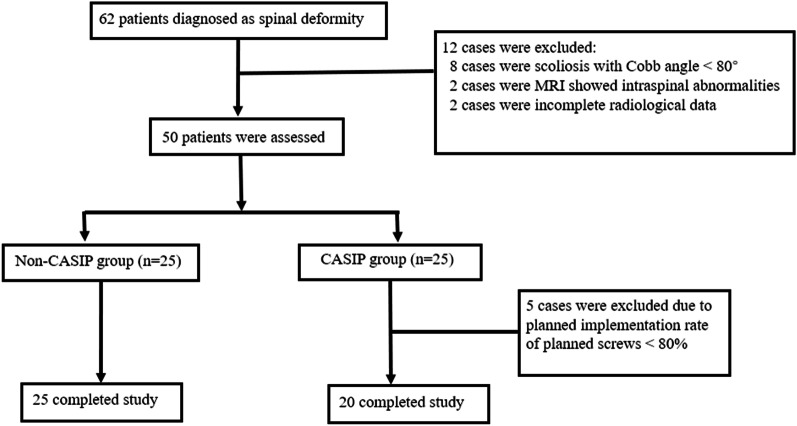
The flowchart

After screening patients, a total of fifty patients were recruited in the study. 25 patients were enrolled in CASIP group and 25 patients were in Non-CASIP group, however, five patients were excluded due to planned implementation rate of planned screws < 80% in CASIP group (The reasons of unplanned implementation included the insufficiency of entry point exposure and additional consideration of the surgeon). Finally, 45 eligible patients were included in the analysis, and 20 were in CASIP group while 25 in Non-CASIP group (Fig. 5). 80% (20/25) of the patients in CASIP group achieved satisfactory planned implementation rate. The detailed demographic data are depicted in Table 1.

**Table 1:** Demographics

	CASIP (n = 20)	Non-CASIP (n = 25)	P
Gender (male/female)	5/15	10/15	
Age (years)	30.5 ± 7.5	33.2 ± 12.8	0.401
Main curve type			
Thoracic	15	19	
Thoracolumbar/lumbar	3	3	
Kyphosis	2	3	
Fusion levels (n)	8.5 ± 1.6	8.0 ± 1.5	0.456
Osteotomy (n)	6	8	0.885
Ponte	2	4	
VCR	4	4	

*BMI* indicates body mass index,* CASIP* computer-assisted screw inserting planning

### Primary outcome

The accuracy of pedicle screw placement in CASIP group was (92.0 ± 5.5) % and (82.6 ± 8.3) % in Non-CASIP group (P < 0.05). In CASIP group, 335 planned screws were assessed, and 26 of them met unsatisfactory grade, and 15 distributed in thoracic spine, 9 in lumbar spine. In Non-CASIP group, 74 screws (433) met unsatisfactory grade. 54 of them were in thoracic spine and 20 in lumbar spine.

### Secondary outcomes

The performance about different radiological outcomes and comparison results were depicted in Tables 2, 3. There was no significant difference between the two groups with regard to EBL and fusion levels. Mean Cobb angle of the main curve was 113.5°±18.2° in CASIP group and 107.6°±13.8° in Non-CASIP group, and focal kyphosis was 62.9° ± 26.5° and 68.8° ± 32.9°, respectively. The main curve was corrected to 54.4° ± 9.8° and 54.7° ± 7.7°, with a correction rate as (51.9 ± 6.5) % and (48.6 ± 8.6) %, respectively, which had no statistical difference. With regard to surgical time, the median was 280.0 (IQR, 260.0–300.0) min in CASIP group and 310 (IQR, 267.5–390.0) min in the other group and the results showed a significant difference (P < 0.05). In CASIP group, no screw puncturing was detected while 11 screws were found in Non-CASIP group, and the incidence of screw puncturing showed significant statistic difference (P < 0.05).

**Table 2:** The comparison of radiological parameters with two groups

	CASIP	Non-CASIP	P
Preoperative			
MC (°)	113.5 ± 18.2	107.6 ± 13.8	0.222
FK (°)	62.9 ± 26.5	68.8 ± 32.9	0.515
TK (°)	42.1 ± 14.6	46.8 ± 15.1	0.294
LL (°)	50.6 ± 9.0	47.0 ± 10.5	0.231
SVA (cm)	2.8 ± 1.4	2.3 ± 1.3	0.282
Postoperative			
MC (°)	54.4 ± 9.8	54.7 ± 7.7	0.900
FK (°)	39.1 ± 7.9	40.1 ± 7.4	0.659
TK (°)	34.3 ± 9.1	37.5 ± 6.3	0.175
LL (°)	42.8 ± 4.6	43.3 ± 8.0	0.778
SVA (cm)	2.1 ± 1.2	1.8 ± 0.9	0.265
Correction rate (%)			
MC	51.9 ± 6.5	48.6 ± 8.6	0.168
FK	33.5 ± 13.9	35.0 ± 16.9	0.762
Surgical time (min)	280.0 (260.0–300.0)	310 (267.5–390.0)	0.034
EBL (ml)	525.0 (450.0–800.0)	500.0 (400.0–775.0)	0.637

*MC* main curve, *FK* focal kyphosis, *EBL* estimated blood loss

**Table 3:** The comparison of screw parameters with two groups

	CASIP (n = 20)	Non-CASIP (n = 25)	P
Screw implantation (n)	16.0 (15.0-18.6)	18.0 (14.5–20.0)	0.526
Screw accuracy (%)	92.0 ± 5.5	82.6 ± 8.3	0.001
Puncturing screw (%)	0 (0–0)	0 (0-6.25)	0.003

## Discussion

Spinal deformity is a three-dimensional pathology, leading to coronal and sagittal plane decompensation, and can result in clinical complaints, neurological deficits, deformity progression, trunk imbalance and cardiopulmonary dysfunction [18–21]. Treatment of severe spinal deformity is one of the ultimate challenges for spine surgeons [22]. Literatures reported the incidence of neurological complications in spinal deformity correction surgeries was 1–27% [5, 9, 23–25], and with regard to new neurological deficits, the incidence was reported as 0.178–9.4%, and 33.6% of it was related to spinal instruments [9, 23]. A systematic review concluded that the rate of screw malposition in scoliosis was 15.7% using CT scan [26]. Hence an accurate and solid screw placement is crucial to achieve a satisfactory and safe correction. Severe spinal deformity has a high risk of screw malposition due to the complex anatomy and vertebra malformation [27, 28]. As reported, Severe spinal deformity was mostly developed without treatment in pediatric period and most of them were idiopathic scoliosis [29]. Majority of severe spine deformity patients suffered from a long-time pathological progress, which caused the pathomorphological changes of the spine including vertebral body or pedicle malformation and thus, we confirmed it could increase the difficulty of spine morphological identification and risks of pedicle screw malposition. With the purpose to improve the accuracy of screw placement in severe spinal deformity, we have explored the feasibility of CASIP in severe spinal deformity. In our series, the accuracy of CASIP group was superior to Non-CASIP group as 92%. We could detect that pedicle screw malposition was mainly distributed in the thoracic spine, which was mostly apical region resulting in extremely rotation and malformation. With CASIP, we could easily recognize whether there existed any malformation as surgical traps for screw placement, and design optimal angle and entry point for inserting. Besides screw related matched parameters were supplied, we also made 3D-printed model as intuitive reference to help the surgeon to insert pedicle screw more accurately during the surgery.

Computer-assisted virtual surgical planning has been reported in hip fracture, femoral fracture, orthopedic oncology and spine surgery [30–33]. Metz et al. reported a case in regard to the application of computer-assisted surgical planning to revision surgery for congenital kyphosis [10]. This technique provided a safe and satisfactory planning in osteotomy and clinical results. Sun et al. reported the usage of this technique in anterior controllable anterior-displacement and fusion surgery for ossification of the posterior longitudinal ligament, and the author demonstrated that the virtual surgical procedure was a feasible and powerful clinically tool for appropriate surgical planning [11]. These researches demonstrated that application of computer-assisted preoperative surgical planning was an optimal tool for surgical planning and desired results were achieved. The Mimics Medical software was a pragmatic and useful tool for CT data reconstruction, 3D printed technique and anatomy measurement. You et al. have reported the application of the software in thoracoscopic anatomical sublobectomy, and demonstrated it was a quick and accurate software for formulating a personalized anatomical surgical plan [34]. We utilized this software to design optimal screw inserting angle, entry point and size for severe spine deformity cases, and this method provided the surgeon an early estimate of the screw and provided matched data combined 3D printed model could help the surgeon quickly identify and solve the difficulty of screw placement.

Parker et al. reported an incidence of 0.22% for PS touching forward major vessels [35]. Despite the rate was rare, it would be catastrophic for patients and surgeons. As we have demonstrated, CASIP could also help us measure the length and width of preset screw so that we could anticipate proper screw size for fixation to avoid aortal or vessel injury forward the vertebra efficiently. To reduce the risk of aortal or vessel injury in the correction surgery, the length of the selected screw was important due to vertebra rotation and malformation [36, 37]. Normally, we would take more intraoperative fluoroscopes to ensure we have selected the optimal screw, however, vertebral rotation did increase the amount of fluoroscopes and decrease the accuracy of the assessment. With preoperative measurement, we could record the accurate size of the screw, and thus, the operation time and radiation for screw position check were saved, and that was the reason we thought about why surgical time was much shorted in CASIP group. Also, the results showed better outcome for puncturing screw in CASIP group.

What we need to pay attention to was that virtual surgical planning was practical but not the same as the real operation. This procedure was operated based on CT scan without regard to muscles and tissue, so it might be different when the patient was anesthetized and placed prone on a surgical table, and matched data of angle and entry point would be critical. The lateral and cephalic angles were relative constant based on the anatomy. Hence, we would be careful to measure and check the angle and entry point for simulated screws to ensure it would be accurate for the surgery. But as we mentioned, 5 patients were excluded due to planned implementation rate was less than 80%. In some of these cases, muscle and tissue exposure were insufficient, and lateral angle applied was not applicable, which decreased the implementation rate.

There are some limitations in the present research. First, this was a pilot study, and the sample size were relatively small. Randomized controlled trials with large samples are warranted. Second, the utilization of the software required an experienced clinical surgeon because specific anatomy need to be recognized, thus more preoperative planning time would be required.

## Conclusions

The application of CASIP in severe spine deformity patients can acquire desirable instruments fixation. With this technique, spine surgeons can make a detailed and personalized screw design preoperatively to achieve solid fixation during the operation.

## Data Availability

The datasets used and/or analyzed during the current study are not publicly available due to the data being confidential; however, they are available from the corresponding author on reasonable request.

## Abbreviations

CASIP: Computer-assisted screw inserting planning
FK: Focal kyphosis
TK: Thoracic kyphosis
LL: Lumbar lordosis
SVA: Sagittal vertical axis
MC: Main curve
BMI: Indicates body mass index
EBL: Estimated blood loss
